# Preparation, structural characterisation, and antioxidant activities of polysaccharides from eight boletes (Boletales) in tropical China

**DOI:** 10.1080/21501203.2022.2069172

**Published:** 2022-05-06

**Authors:** Run Tian, Hui Chai, Jun-Qiang Qiu, Zhi-Qun Liang, Hui-Jing Xie, Yong Wang, Nian-Kai Zeng

**Affiliations:** a Key Laboratory of Tropical Translational Medicine of Ministry of Education, School of Pharmacy, Hainan Medical University, Haikou, China; b College of Science, Hainan University, Haikou, China

**Keywords:** *Crocinoboletus rufoaureus*, antioxidant activity, physicochemical property, polysaccharides, boletes

## Abstract

Polysaccharides in boletes (Boletales) are economically significant to both function food and medicinal industries. The polysaccharides were extracted from the fruit bodies of eight boletes, namely, *Aureoboletus longicollis, Butyriboletus hainanensis, Crocinoboletus rufoaureus, Hemioporus japonicus, Neoboletus infuscatus, Neoboletus obscureumbrinus, Tylopilus otsuensis, Xanthoconium fusciceps*, which were collected from tropical China; their physicochemical properties and antioxidant activities were characterised and evaluated, respectively. The results revealed that the polysaccharides among the eight boletes were mainly composed of glucose, mannose, and galactose, with a broad molecular weight range, and contained a pyranose ring revealed by FT-IR and NMR spectral analyses. Many factors such as different species of boletes, geographic conditions, molecular weight, configuration, and monosaccharide content may affect the antioxidant power of polysaccharides, simultaneously, instead of one single factor. The antioxidant activities of the polysaccharides were measured according to *in vitro* assays of DPPH scavenging, superoxide anion scavenging, and ferrous ion reducing tests. The polysaccharide of *C. rufoaureus* has greatly superior antioxidant activity and it could serve as potential functional food or medicine.

## 1. Introduction

Polysaccharides, one of the most abundant constituents in mushrooms, have attracted much attention for their special physicochemical properties and potential activities (Zhang et al. 2018). For example, polysaccharides derived from mushrooms, like ganoderan, lentinan, and grifolan have been investigated as the focus of research (Wu 2018; López-Legarda et al. 2020; Yang et al. 2021). Boletes (Boletales), one important group of mushrooms, have been reported recently, showing high species diversity (Yang et al. 2008; Zhang et al. 2011; Zeng et al. 2015, 2018b; Liang et al. 2016; Wu et al. 2019; Jiang et al. 2021). Polysaccharides extracted from boletes also display a wide range of bioactivities, especially strong antioxidant activities (Sun et al. 2016a; Yang et al. 2008; Zhang et al. 2011, 2018). However, only a few polysaccharides from boletes were investigated (Sun et al. 2016; Wu et al. 2016; Su et al. 2018; Xiao et al. 2019; Meng et al. 2021), polysaccharides from more boletes should be noted.

Recently, eight boletes were collected from tropical China, crude polysaccharides were extracted from the fruit bodies of eight boletes. The structures of the crude polysaccharides were characterised according to high-performance liquid chromatography (HPLC) and Fourier-transform infrared spectroscopy (FT-IR), respectively; their antioxidant properties were studied using *in vitro* assays of DPPH scavenging, superoxide anion scavenging, and ferrous ion reducing tests. The purposes of this study are: (i) to elucidate the structures of polysaccharides and explore their antioxidant activities from eight boletes; and (ii) to uncover more medicinal boletes from China.

## 2. Materials and methods

### 2.1. Materials and chemicals

**Figure 1.**
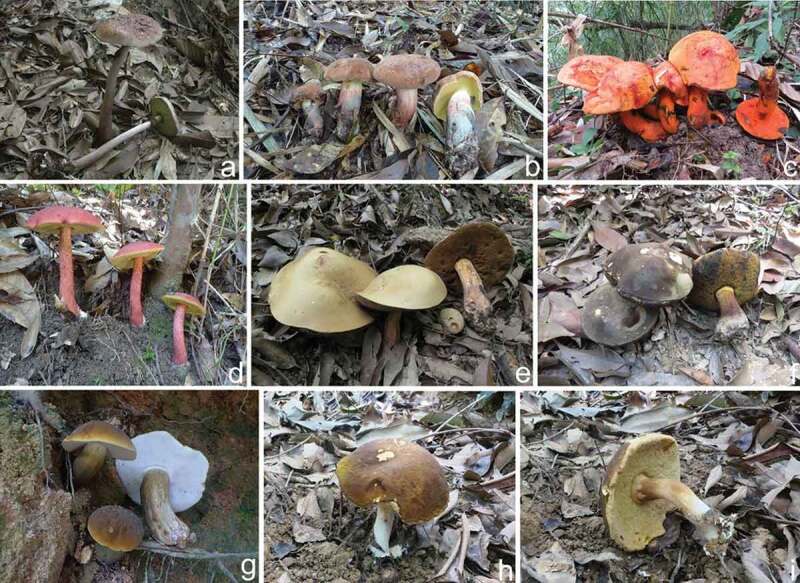
Basidiomata of eight boletes. (a). *Aureoboletus longicollis* (FHMU398). (b). *Butyriboletus hainanensis* (FHMU2410). (c). *Crocinoboletus rufoaureus* (FHMU1975). (d). *Hemioporus japonicus* (FHMU887). e. *Neoboletus infuscatus* (FHMU3372). (f). *N. obscureumbrinus* (FHMU2052). g. *Tylopilus otsuensis* (FHMU914). (h–i). *Xanthoconium fusciceps* (FHMU4759). Photos by N.K. Zeng.

Fruit bodies of eight boletes collected from Hainan Island, a tropical area of China, are demonstrated in Figure 1. The voucher specimens were deposited in the Fungal Herbarium of Hainan Medical University, Haikou City, Hainan Province, China (FHMU). Techniques for species identification using morphological and molecular phylogenetic analyses followed those in Zeng et al. (2015, 2018), Liang et al. (2016), Chai et al. (2019), Wu et al. (2019), Jiang et al. (2021), and references therein. Five sequences [2 of nuc 28S rDNA D1-D2 domains (28S) (MW826956, OK316964), 2 of nuc rDNA region encompassing the internal transcribed spacers 1 and 2, along with the 5.8S rDNA (ITS) (MW830220, OK298390), and 1 of the translation elongation factor 1-α gene (*TEF1*) (MW925783)] were newly generated and deposited in GenBank. The results of species identification indicated that the eight boletes were *Aureoboletus longicollis* (Ces.) N.K. Zeng & Ming Zhang, *Butyriboletus hainanensis* N.K. Zeng, Zhi Q. Liang & S. Jiang, *Crocinoboletus rufoaureus* (Massee) N.K. Zeng, Zhu L. Yang & G. Wu, *Hemioporus japonicas* (Hongo) E. Horak, *Neoboletus infuscatus* N.K. Zeng, S. Jiang & Zhi Q. Liang, *N. obscureumbrinus* (Hongo) N.K. Zeng, H. Chai & Zhi Q. Liang, *Tylopilus otsuensis* Hongo, and *Xanthoconium fusciceps* N.K. Zeng, Zhi Q. Liang & S.Jiang, respectively. The abbreviations of *A. longicollis, But. hainanensis, C. rufoaureus, H. japonicas, N. infuscatus, N. obscureumbrinus, T. otsuensis*, and *X. fusciceps* in this work were Al., Bh., Cr., Hj., Ni., No., To., Xf., respectively.

1,1-Dipheneyl-2-picrylhydrazyl (DPPH) was purchased from Tokyo Kasei Kogyo Co. Ltd. (Shanghai, China); 2,6-ditert-butyl-4-methylphenol (BHT) and 1-phenyl-3-methyl-5-pyrazolone (PMP) were bought from Shanghai Macklin Biochemical Technology Co. Ltd (Shanghai, China); ascorbic acid (ASC) was obtained from Beijing Solarbio Science & Technology Co., Ltd. (Beijing, China); standard monosaccharide samples were all from Sigma-Aldrich (Germany); other chemicals used were of analytical grade, and mainly produced by Xilong Scientific Co., Ltd. (Guangdong, China).

### 2.2. Preparation of water-soluble crude polysaccharides

The dried samples were ground into fine powders (40 meshes). The crude polysaccharides were prepared as the previously reported method (Liu et al. 2016), with some minor modifications. The powders (10 g) were subjected to macerating with 95% ethanol (v/v) overnight. The residues were dried and then extracted with hot water at 85°C for 3 h and then repeated twice. The filtrates were combined and evaporated to 20 mL in vacuum. The proteins in the solution were eliminated by Sevag reagent. Then, the deproteinized liquid was dialysed against tap water for 24 h and distilled water for 12 h. The dialysate was concentrated and then precipitated with 4 volumes of ethanol-water solution and centrifuged, and finally lyophilised to obtain the crude polysaccharides. The crude polysaccharide content was determined using the phenol-sulphuric acid spectrophotometric method according to the established method (Kaewnarin et al. 2020).

### 2.3. Molecular weight determination

Molecular weight (MW) distributions of the crude polysaccharides were measured by referring to the reported method (Zhang et al. 2018). Each sample (10 mg) was dissolved in 5 mL of deionised water to obtain a sample solution at the concentration of 2 mg/mL. The solution was stirred with a magnetic stirrer for 30 min before analysis. Then the MW distributions were determined using high-performance gel permeation chromatography (HPGPC) matching a TSK-GEL G5000 PW column (7.8 × 300 mm, Tosoh Corp, Japan) on an Agilent 1100 HPLC system equipment equipped with a Waters 2410 refractive index detector, and eluted with ultrapure water at a flow rate of 0.8 mL/min. The column temperature was maintained at 30°C. A 20 μL of polysaccharide solution was injected in each run. Dextran standards in the range of 3.5 to 670 kDa were used to establish the standard curve. The results were processed and analysed using Breeze Software.

### 2.4. Monosaccharide composition

The monosaccharide compositions of the polysaccharides from eight boletes were analysed using PMP-HPLC according to a previously reported method (Ni et al. 2014), with minor modifications. Each dried crude polysaccharide sample (10 mg) was hydrolysed using 2 mL of 2 mol/L trifluoroacetic acid (TFA) at 110°C for 2 h. After being cooled down to room temperature, 1 mL of solution was mixed with 1 mL of methanol, then the mixture was dried by nitrogen at 70°C in a water bath. The procedure was repeated twice to eliminate the excessive acid. One millilitre of sodium hydroxide solution (0.3 M) was subsequently added to dissolve it completely.

Prepare a mixed monosaccharide solution containing mannose, rhamnose, glucosamine, glucose, galactose, xylose, arabinose, and fucose at a concentration of 0.4 mg/mL, then mix it with sodium hydroxide solution (0.6 M) at an equivalent volume. A 400 μL of PMP methanol solution was reacted with 400 μL of the mixture solution at 70°C for 2 h. After the reaction solution was cooled down to room temperature, 400 μL dilute hydrochloric acid (0.3 M) was then added to neutralise the solution, and its pH value was kept between 6 and 7. Then, 1.2 mL water was added and partitioned with an equivalent volume of chloroform. The procedure was repeated twice. The water phases were combined and filtered with microporous membrane (0.45 μm). The polysaccharide hydrolysates were derivatised under the same conditions as PMP.

The monosaccharide composition analysis was performed through a C_18_ column (250 mm × 4.6 mm, 5 μm, Agilent Corp., USA) on an Agilent 1100 HPLC with a DAD detector. The mobile phase was sodium phosphate buffer (100 mM, pH 6.7) (A) – acetonitrile (B). The gradient elution mode was as follows: 0–9 min, 86%A→83%A; 9–28 min, 83%A→78%A; 28–29 min, 78%A→50%A; 29–31 min, 50%A; 31–32 min, 50%A→86%A; 32–36 min, 86%A. The detection wavelength was set at 250 nm, the column temperature was maintained at 30°C, and the injection volume was 10 μL aliquot for each run at a flow rate of 1 mL/min.

### 2.5. FT-IR spectral analysis

FT-IR spectra of crude polysaccharides were recorded on a Fourier-transform infrared spectrometer (Nicolet iS5 FT-IR, Thermo Fisher Corporation, USA) in the range of 400–4000 cm^−1^ at room temperature. Each polysaccharide was incorporated with KBr powder at a ratio of 1: 100, pressed into a pellet, and then scanned for FT-IR measurement.

### 2.6. NMR spectral analysis

The polysaccharides were dried for 72 h in a vacuum dryer filled with silica gel and then dissolved in 0.6 mL deuterium oxide (D_2_O). The ^1^H NMR spectra of eight samples were performed on a JEOL 400 MHz NMR spectrometer. All NMR data were analysed using MestReNova software.

### 2.7. In vitro antioxidant activities

#### 2.7.1. DPPH radical scavenging activity

The DPPH radical scavenging assay of the eight samples was investigated according to the report by (Kaewnarin et al. 2020), with minor modifications. Polysaccharide samples (10 mg) were dissolved in distilled water and then diluted to 10 mL to obtain the sample solution at a concentration of 1 mg/mL. The antioxidant activity at various doses of polysaccharides was evaluated using DPPH in a 96-well plate. And the absorbance at 517 nm was measured with a pan-wavelength automatic microplate reader (Spectra MAX1900, Meigu Molecular Devices Co. Lit., Shanghai, China). Lower absorbance indicates high free radical scavenging activity. The positive controls were BHT and ASC, the negative and blank control was the reaction solution without polysaccharide and distilled water. Each test was repeated three times in parallel. The DPPH radical scavenging rate was determined as follows:

$$DPPH\;radical\;scavenging\;rate\left(\%\right)=\frac{A_{0}-A_{i}}{A_{0}}\times 100$$

Where *A_i_* was the absorbance of the solution containing a sample; *A_0_* was the absorbance of the mixture of DPPH solution and distilled water. The EC_50_ value, which was measured by establishing DPPH radical scavenging ability versus polysaccharide concentration, denoted the concentration of polysaccharide producing 50% inhibition.

#### 2.7.2. Superoxide anion scavenging ability

The scavenging ability of superoxide radical was determined in accordance with the previously described method (Vamanu 2012), with some minor modifications. In brief, each polysaccharide was dissolved in water, and a series of sample solutions were diluted to various concentrations. The ability of the investigated fraction to scavenge superoxide radical was determined with various doses of samples (0.1–0.5 mg/mL), which were mixed in the 96-well plate and OD values were measured at 325 nm. The positive controls were BHT and ASC, the blank control was the reaction mixture without polysaccharides. Each test was repeated three times in parallel. The scavenging rate was also calculated by the following equation:

$$Superoxide\;anion\;scavenging\;rate\left(\%\right)=\frac{A_{0}-\left(A_{i}-A_{j}\right)}{A_{0}}\times 100$$

Where *A_0_* was the absorbance of blank control, *A_i_* represented the absorbance of the solution including a sample, and *A_j_* stood for the absorbance of the pyrogallol solution substituted by pyrogallol-HCl solution.

#### 2.7.3. Ferrous ion reducing power

The power of the polysaccharide to reduce ferrous ions was detected using the method of Vamanu E (Vamanu 2012), with minor modifications. The test solution consisted of the mixture of 1 mL of sodium phosphate buffer (pH 6.6), 1% potassium hexacyanoferrate solution (w/v), and test sample solution. The polysaccharide samples with different concentrations (0.2–1.0 mg/mL) were mixed in the 96-well plate and absorbance was calculated at 700 nm with a pan-wavelength automatic microplate reader (Spectra MAX1900, Meigu Molecular Devices Co. Lit., Shanghai, China). Higher absorbance was indicative of greater reducing ability. BHT and ASC were used as the positive controls and the reaction solution without polysaccharides was served as the blank control.

### 2.8. Statistical analysis

All experiments were carried out in triplicate and the results were shown as means ± standard deviation. Statistical significance was assessed using one-way analysis of variance (ANOVA) followed by Tukey´s tests. A value of *P* < 0.05 was judged statistically significant. Pearson correlation and multiple linear regression analysis were applied to investigate the relationships between polysaccharide characteristics and antioxidant activities. All analyses of data from the experiment were processed by SPSS and Origin software.

## 3. Results

### 3.1. Assay of crude polysaccharides

The glucose standard curve was plotted with the concentration values of the standard solution as the horizontal coordinates and the OD values at 490 nm as the vertical coordinates. The regression equation of the standard curve was obtained as follows: y = 7.3714x + 0.0037, *R*^2^ = 0.9994. The result indicated that the standard curve showed good linearity in the concentration ranging from 0 to 0.07 mg/mL.

Eight crude polysaccharides were obtained through defatting, aqueous extraction, deproteinization, and ethanol precipitation, consecutively. The yield of the polysaccharides was calculated according to the standard curve. The yield of crude polysaccharides of the eight samples followed the order of Cr. < Xf. < Bh. < Hj. < Al. < Ni. < To. < No., which was 29.38 ± 1.72%, 32.46 ± 1.1%, 35.62 ± 0.55%, 37.25 ± 1.51%, 37.70 ± 1.72%, 48.83 ± 2.21%, 54.53 ± 2.72%, and 60.50 ± 0.95%, respectively. The highest yield of crude polysaccharides, which was 60.50%, was obtained from *N. obscureumbrinus* (sample No.), followed by the *T. otsuensis* (sample To.), which was 54.53%. However, the crude polysaccharide yield of *C. rufoaureus* (sample Cr.) was the lowest among the eight boletes, which was only 29.38%. Among these samples, there was no significant difference in the crude polysaccharide yield of *A. longicollis* (sample Al.), *B. hainanensis* (sample Bh.), and *H. japonicus* (sample Hj.), all of which ranged from 35% to 37%.

### 3.2. Molecular weight determination

Weight-average molecular weight (Mw), number-average molecular weight (Mn), and the Mw/Mn of polysaccharide fractions from eight boletes are shown in Table 1, and the average Mw of crude polysaccharides isolated from these boletes was different. The polysaccharide of *H. japonicus* (sample Hj.) exhibited the highest Mw among the eight boletes (Mw = 31,665 Da), while *C. rufoaureus* (sample Cr.) had the lowest Mw (Mw = 5286 Da). The crude polysaccharides from eight boletes showed an Mw range from 5.29 × 10^3^ Da to 3.17 × 10^4^ Da, and the average Mw of the polysaccharides from these boletes were similar, ranging from 10^3^ Da to 10^4^ Da.

**Table 1.** The molecular weight of crude polysaccharides from eight boletes.

Sample	Al.	Bh.	Cr.	Hj.	No.	Ni.	To.	Xf.
Mw (Da)	22,106	5306	5286	31,665	5497	9461	9605	6237
Mn (Da)	13,470	3414	3684	14,182	3786	8378	8889	4191
Mw/Mn	1.58	1.51	1.45	2.07	1.38	1.11	1.07	1.35

### 3.3. Monosaccharide composition

**Figure 2.**
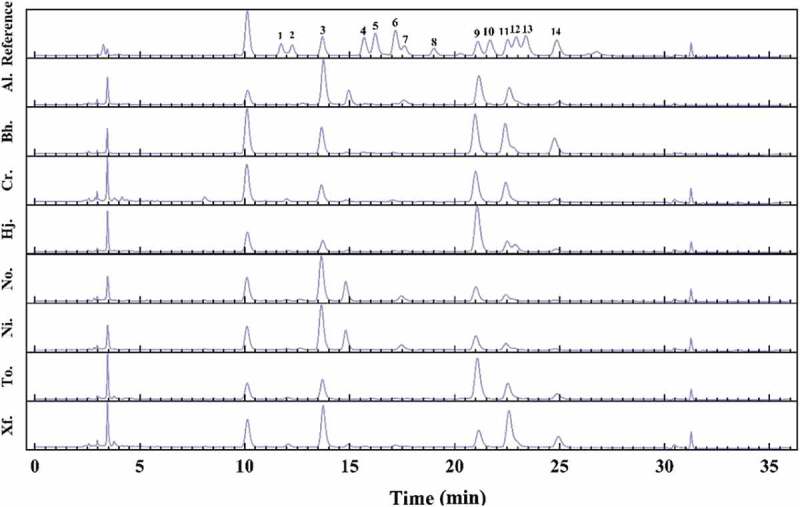
Fingerprint chromatograms of the monosaccharide compositions of crude polysaccharides from eight boletes and fourteen kinds of reference monosaccharides. 1. GulUA. 2. ManUA. 3. Man. 4. Rib. 5. Rham. 6. GlcN. 7. GlcUA. 8. GalUA. 9. Glc. 10. GalN. 11. Gal. 12. Xyl. 13. Ara. 14. Fuc.

The monosaccharide composition and HPLC chromatograms of the crude polysaccharides from the eight boletes are presented in Table 2 and Figure 2, respectively. The crude polysaccharides from the investigated boletes mainly consisted of mannose, glucosamine, glucose, galactose, xylose, and fucose; mannose, glucose, and galactose were the most abundant monosaccharides of the crude polysaccharides, while the content of glucose was the highest among them.

**Table 2.** The monosaccharide composition of crude polysaccharides from eight boletes.

Sample	Monosaccharide composition (mg/g)							
	Man	Rham	GlcN	Glc	Gal	Xyl	Ara	Fuc
Al.	87.71	0.87	1.55	77.26	41.82	5.03	0.30	7.57
Bh.	51.84	1.74	1.92	109.27	77.07	9.51	0.00	35.88
Cr.	25.19	0.64	2.10	64.13	38.79	1.22	0.00	6.02
Hj.	36.22	1.03	1.77	201.87	40.22	23.56	0.00	11.04
No.	158.19	0.00	0.25	67.59	29.25	5.50	0.00	5.06
Ni.	69.15	0.60	1.75	133.49	93.64	15.27	0.27	32.62
To.	60.93	1.84	1.76	170.85	63.70	2.37	1.01	19.57
Xf.	63.62	0.00	2.40	35.73	72.05	6.49	0.68	20.36

The monosaccharide compositions of the crude polysaccharides from eight boletes were different, for example, the arabinose in the crude polysaccharide of *H. japonicus* was not detected, with glucose content being the most abundant (201.87 mg/g); polysaccharide in *N. obscureumbrinus* had the highest content of mannose, with rhamnose and arabinose not detected; additionally, arabinose was not detected in *But. hainanensis, C. rufoaureus*, and *H. japonicus*, rhamnose was not found in *X. fusciceps*, and neither glucose nor rhamnose was detected in *N. obscureumbrinus.*

### 3.4. FT-IR spectral analysis

**Figure 3.**
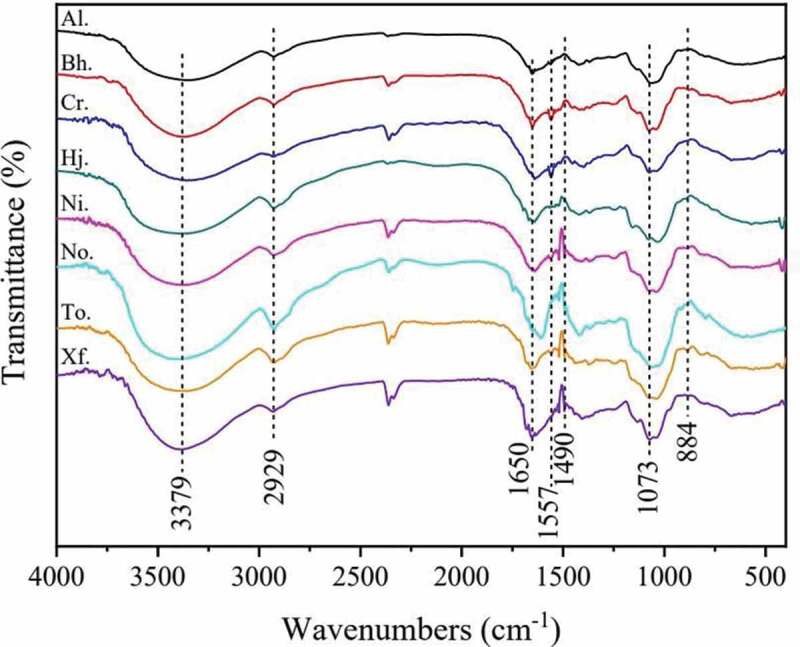
FT-IR spectra of crude polysaccharides from eight boletes.

The experimental result of the FT-IR of crude polysaccharides from eight boletes exhibited in Figure 3. As seen in Figure 3, the spectra of eight crude polysaccharides were almost similar to each other, but there were some differences in the absorption peaks and their positions, which could be attributed to different compositions and content of monosaccharides from the eight boletes. All samples suggested a broad stretching peak at 3370 cm^−1^ typical of hydroxyl groups as well as a C-H absorption peak at 2930 cm^−1^, which was thought to be the characteristic of polysaccharides (Liu et al. 2012). Besides, the absorption band 1800–1500 cm^−1^ was accounted for the stretching vibration of the bound water. The peaks near 1600 cm^−1^ could be due to symmetrical deformation vibration of the carbonyl group of the polysaccharides, while the centre of the peaks at 1500 cm^−1^ may be correlated to the symmetrical formation vibration of carbonyl group. Moreover, the absorption peak in the range of 1200–1000 cm^−1^ suggested the existence of the pyranose ring among all samples. In previous studies, the peak near 798 cm^−1^ exhibited the existence of an alpha configuration, and the peak around 888 cm^−1^ showed the presence of a *β*-configuration (Liu et al. 2012; Fan et al. 2018). From what has been analysed above, we could conclude that all samples from the eight boletes mainly contain the *β*-configuration, which was in line with those of two other boletes revealed by previous studies, viz. *L. rugosiceps* and *Boletes* sp. (Yang et al. 2008; Li et al. 2020).

### 3.5. NMR analysis

**Figure 4.**
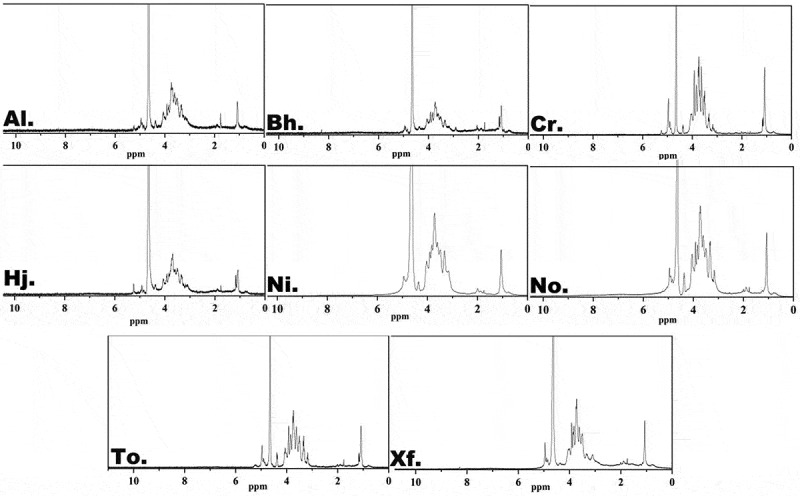
^1^H NMR spectra of crude polysaccharides from eight boletes.

The ^1^H NMR spectra of polysaccharides from eight boletes are displayed in Figure 4. The crowded and narrow regions of eight samples ranged from 3.0 to 5.5 ppm were typical signals of polysaccharides, which was consistent with our studies (Wan et al. 2022). In general, the anomeric hydrogen signals of polysaccharides were generated at δ 4.3–5.9, and the anomeric proton signals at δ 4.47–5.0 were responsible for the *β*-pyranose unit (Corsaro et al. 2005). Besides, some of the absorption peaks above 5 ppm could relate to the purity of crude polysaccharides and the existence of *α*-pyranose. And the ^1^H NMR spectra suggested that *β*-pyranose existed in eight polysaccharides, which was in line with the analysis of the FT-IR spectra. Then, the results with other polysaccharides in NMR spectra were very similar to these (Cheng et al. 2020; Li et al. 2020; Wan et al. 2022). Li et al. (2020) suggested the ^1^H NMR spectrum of LRP-1 was crowded in narrow regions ranging from 3.1 to 4.5 ppm. Furthermore, some absorption peaks around 1 ppm could be associated with the existence of the methylated polysaccharides.

### 3.6. Antioxidant activities in vitro

#### 3.6.1. DPPH radical scavenging activity

**Figure 5.**
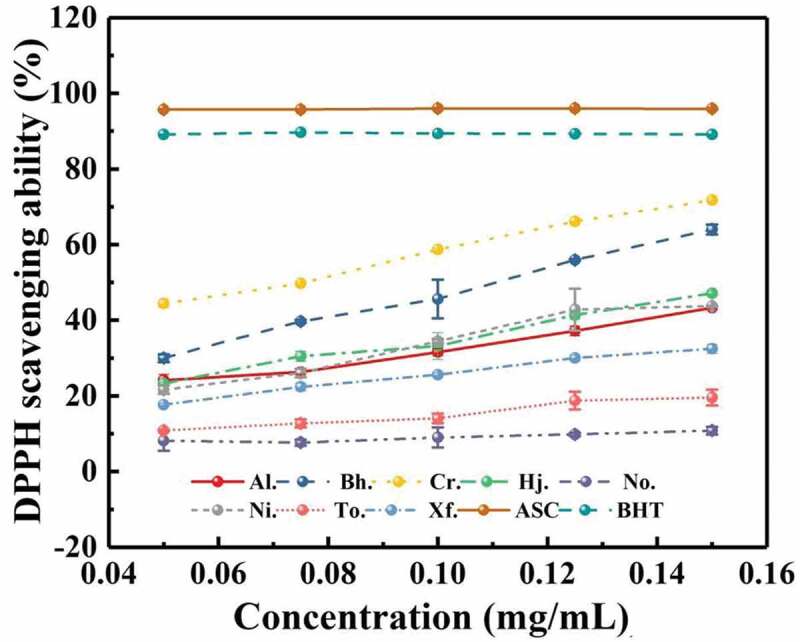
Scavenging ability of crude polysaccharides from eight boletes on DPPH.

The scavenging ability of all samples from eight boletes on hydroxyl-free radical is summarised in Figure 5. In the concentration range of 0.05–0.15 mg/mL, DPPH scavenging ability was improved with increasing concentration of these crude polysaccharides, although the DPPH scavenging abilities of BHT and ASC were significantly higher than those of eight boletes. In this study, DPPH scavenging abilities from different polysaccharides samples were different. The polysaccharide from *C. rufoaureus* presented the strongest DPPH scavenging ability, followed by that of *But. hainanensis*, and followed by *T. otsuensis*, and followed by *A. longicollis, H. japonicus*, and *N. infuscatus*, which had similar scavenging DPPH ability. In addition, *N. obscureumbrinus* had the lowest scavenging ability. The scavenging ability of the crude polysaccharides from eight boletes ranked as Cr. (71.75%) > Bh. (64%) > Hj. (47.16%) > Ni. (43.75%) > Al. (43.31%) > Xf. (32.49%) > To. (19.56%) > No. (10.81%), when the concentration of the crude polysaccharides came to 0.15 mg/mL.

#### 3.6.2. Superoxide anion scavenging ability

**Figure 6.**
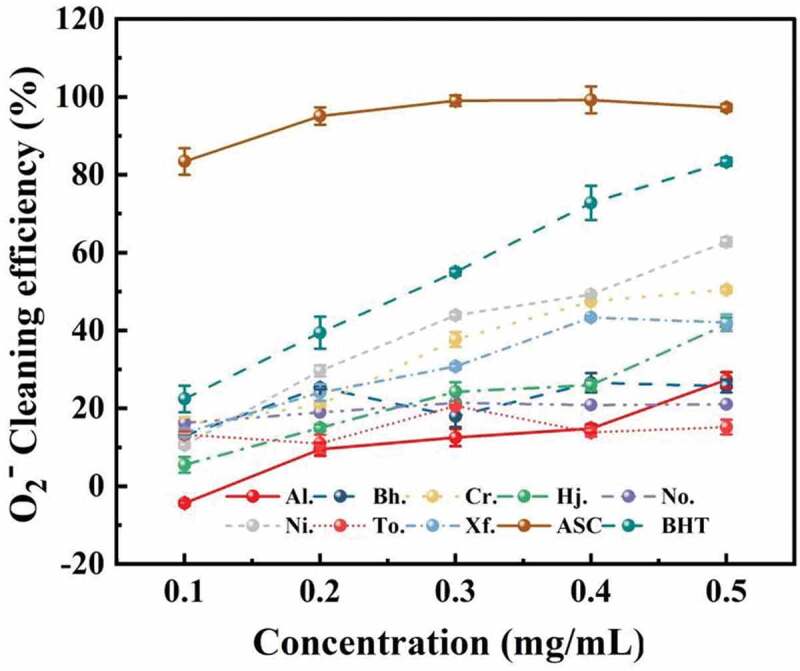
The scavenging ability of crude polysaccharides from eight boletes on superoxide radical.

Previous studies have revealed that antioxidant activities of boletes have a relationship with scavenging ability on superoxide radicals (Zhang et al. 2011). The results of the superoxide anion scavenging ability of the samples is exhibited in Figure 6. In the concentration range of 0.1–0.5 mg/mL, the scavenging ability of polysaccharides from most boletes improved with the concentration increased, except for *But. hainanensis* and *T. otsuensis*. When the concentration of polysaccharide came to 0.5 mg/mL, *N. infuscatus* and *C. rufoaureus* had greater scavenging ability on superoxide radical than other samples, but weaker than that on the positive controls (BHT and ASC), with 62.78% and 50.52%, respectively. When the concentration of crude polysaccharide from eight boletes reached 0.5 mg/mL, the scavenging ability on superoxide scavenging decreased in the order of Ni. (62.78%) > Cr. (50.52%) > Xf. (42.04%) > Hj. (41.56%) > Al. (27.31%) > Bh. (25.72%) > No. (21.05%) > To. (15.19%), indicating some differences compared with those in the DPPH scavenging activity assay.

#### 3.6.3. Ferrous ion reducing power

**Figure 7.**
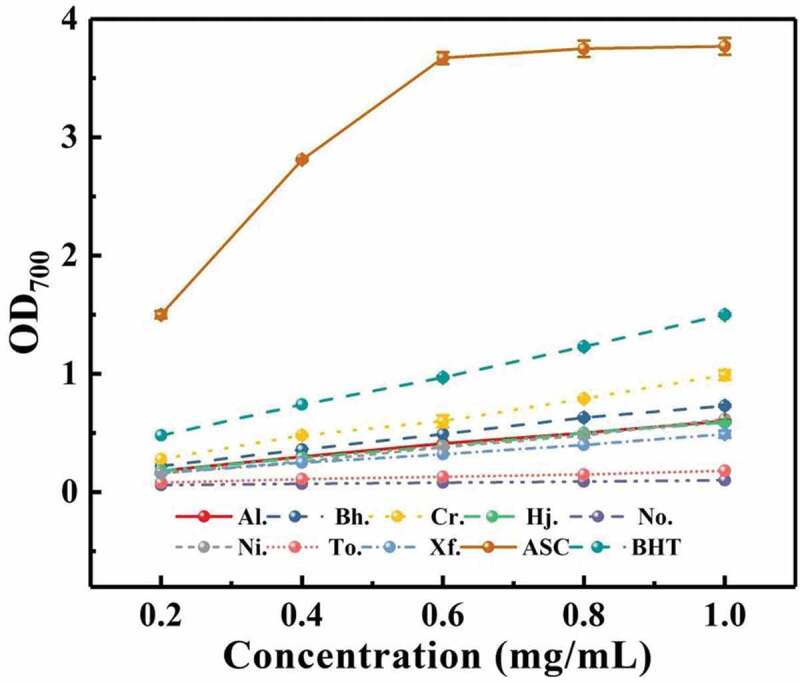
Reducing power of crude polysaccharides from eight boletes.

The result of the ferrous ion reducing power of polysaccharides can be seen in Figure 7. The result clearly presented that the reducing ability of eight samples was in concentration-dependent manner in the range of 0.2–1 mg/mL, higher OD_700_ value meant stronger reducing ability of samples from boletes. Among these samples, *C. rufoaureus* presented the greater reducing power than the other crude polysaccharide samples, with the OD_700_ values being 0.99 at the concentration of 1 mg/mL, although the OD_700_ value of *C. rufoaureus* was still weaker than that of positive control; while ferrous ion reducing tests indicated that the polysaccharide extracted from *N. obscureumbrinus* had the least significant effect compared with other samples. When the concentration of polysaccharide came to 1 mg/mL, the reducing power (OD_700_ value) of eight boletes ranked as Cr. (0.99) > Bh. (0.73)> Ni. (0.62) > Al. (0.60) > Hj. (0.59) > Xf. (0.49) > To. (0.18) > No. (0.10). Experimental results were similar to those of previous DPPH radical scavenging assays, the extracts from *C. rufoaureus* showed stronger reducing activities than those of the other seven polysaccharide extracts.

## 4. Discussion

Although the antioxidant activity of polysaccharides produced by boletes including *B. aereus* Bull., *B. edulis* Bull., *B. speciosus* Frost, *B. violaceo-fuscus* W.F. Chiu, *Suillus bovinus* (L.) Roussel, and *S. luridus* (Schaeff.) Richon & Roze was investigated by Ding et al. (2012), Luo et al. (2012), and Zhang et al. (2018), more boletes used for antioxidant should be investigated for the structural characterisations and activities of polysaccharides have been proved to be different for different species (Jiao et al. 2010; Ren et al. 2014; Zheng et al. 2014; Meng et al. 2015; Sun et al. 2016b; Su and Li 2020; Li et al. 2020). In the present study, the structural characterisations and activities of polysaccharides of eight boletes from Hainan, tropical area of China were never investigated before.

The previous study showed that average molecular weight of some polysaccharide fractions isolated from *B. edulis* and *B. speciosus* were ranging from 10^3^ Da to 10^4^ Da (Luo et al. 2012), which was consistent with the results of eight boletes in our study. However, Sun et al. (2016), Zhang et al. (2018); Li et al. (2020) reported that the average molecular weights of crude polysaccharides extracted from boletes including *B. aereus, B. edulis, B. speciosus, B. violaceo-fuscus, Leccinellum rugosiceps* (Peck) C. Hahn [basionym: *Leccinum rugosiceps* (Peck) Singer], *Rubroboletus sinicus* (W.F. Chiu) Kuan Zhao & Zhu L. Yang (basionym: *B. sinicus* W.F. Chiu), *S. bovinus*, and *S. luridus* were 10^5^ to 10^7^ Da. Recently, Ren et al. (2014) demonstrated that polysaccharides with a low MW or a *β*-configuration in the pyranose form displayed a higher antioxidant activity. Xie et al. (2016) demonstrated that polysaccharides with a larger molecular weight had a weaker antioxidant activity than lower MW polysaccharides due to the steric hinderance. However, Zheng et al. (2014) presented that the extracts isolated from the submerged culture of *B. aereus*, with the higher molecular weight, showed remarkably great antioxidant power among the three polysaccharide fractions. Simultaneously, in the present study, *C. rufoaureus* with a lower molecular weight (5.29 × 10^3^), also exhibited a stronger DPPH scavenging power and ferrous ion reducing ability than other samples with a higher molecular weight while *N. infuscatus* with a higher molecular weight (9.46 × 10^3^) among the samples, also possessed best superoxide anion scavenging ability. Our result was agreed well with Zhang et al. (2018) that effect of polysaccharide Mw on antioxidant activity is maintained unclear.

Then, our resulting data coincided with those of previous studies, which exhibited that mannose, glucose, and galactose were also the most abundant monosaccharides in *B. edulis* and *R. sinicus* (Jiao et al. 2010; Sun et al. 2016b); *B. aereus, B. edulis, B. violaceo-fuscus, S. bovinus*, and *S. luridus* in which glucose was the most abundant monosaccharide constituents of the polysaccharides (Zhang et al. 2018), while *A. longicollis, X. fusciceps*, and *N. obscureumbrinus* with mannose being the most abundant were exhibited in our study. According to Sun et al. (2016), BSP-2b, a novel polysaccharide from *R. sinicus*, mainly consisted of mannose, glucuronic acid, glucosamine glucose, and galactose, however, glucuronic acid and glucosamine glucose were not found in our study. The monosaccharide compositions of crude polysaccharides from eight boletes in our study were different from those revealed in previous studies. Moreover, Zhang et al. (2018) reported that the scavenging abilities for the DPPH radical exhibited an obvious correlation with the arabinose and the galactose content of the polysaccharides. According to the linear regression analysis, there were no obvious correlations between the monosaccharide content and the DPPH scavenging percentages of the polysaccharides in our study (*P* > 0.05). Su and Li (2020) also demonstrated that there were no significant correlations between the monosaccharide content and DPPH scavenging ability of polysaccharides, but significant correlations between monosaccharide content and molecular weight. In addition, Meng et al. (2015) put forward a different view and reported that the antioxidant activity of polysaccharides showed correlation with mannose content (*P* < 0.01) and glucose content (*P* < 0.05), instead of galactose content (*P* > 0.05). Thus, the ability of monosaccharide components to affect antioxidant activity may vary from species to species.

Additionally, previous studies indicated that other factors except for MW, including molecular configuration, sulphation, and so on, also played a significant role in the antioxidant activities of polysaccharides (Wang et al. 2017). For example, *β*-configuration of polysaccharides tended to form a stable triple-helical structure, which had biological activities such as antitumor, antiviral, and antioxidant activities, whereas *α*-configuration usually has no biological activities (Li and Wang 2002). And thus, the *β*-configuration of the glucose molecule in a helix structure was considered to be significant for the biological and pharmacological activity (Mizuno 1999). In the present study, the results revealed that the polysaccharides among the eight boletes contained a pyranose ring revealed by FT-IR and NMR spectral analyses. The polysaccharides extracted from *C. rufoaureus*, with a *β-*configuration in the pyranose form, suggested the greater scavenging power on DPPH and reducing ability. Thus, we can speculate that the antioxidant activity of boletes may be due to the presence of *β*-configuration.

In previous investigations, the antioxidant properties of polysaccharides could be associated with their structural characteristics such as monosaccharide composition, molecular weight distribution and degree of branching or sulphation (Lo et al. 2007; Sun et al. 2009; Lo et al. 2012). According to the all the above, we speculated that many factors such as species, molecular weight, configuration and monosaccharide content, may affect antioxidant power of the crude polysaccharides, simultaneously, instead of one single factor.

## 5. Conclusions

All crude polysaccharides were extracted from the fruit bodies of eight boletes after defatting, aqueous extraction, deproteinization, and ethanol precipitation, subsequently. Eight polysaccharides from boletes were characterised by PMP-HPLC, HPGPC, FT-IR spectroscopy, and NMR spectra analysis, and their antioxidant abilities were consecutively evaluated. The results demonstrated that the crude polysaccharides from eight boletes in tropical China were mainly composed of mannose, glucose, and galactose, and possessed the beta configuration in the pyranose form; yield, Mw, and the monosaccharide composition of the crude polysaccharides from different boletes were greatly different in the present study. Moreover, the antioxidant assay indicated that *C. rufoaureus* presented significantly great antioxidant activities among those of eight boletes, and exhibited superior potential in the application of the natural antioxidant activity.
